# When Affective Relation Weighs More Than the Mug Handle: Investigating Affective Affordances

**DOI:** 10.3389/fpsyg.2020.01928

**Published:** 2020-08-21

**Authors:** Marta Caravà, Claudia Scorolli

**Affiliations:** Department of Philosophy and Communication Studies, University of Bologna, Bologna, Italy

**Keywords:** affective affordances, enactivism, embodied cognition, level and modality of integration, frequency of exposure, affective practices, kinematic analysis, temporal dynamics

## Introduction

Enactive and embodied approaches to cognition are becoming increasingly interested in the affective dimension of human experience (Varela and Depraz, 2005; Colombetti, 2007, 2014; Colombetti and Thompson, 2008; Di Paolo and De Jaegher, 2015; Gallagher and Varga, 2015; Gallagher and Allen, 2016; Scorolli, 2019). Consistently, this issue has been addressed in empirical research, which is paying growing attention to the affective quality of social contexts by addressing motor simulations (Bastiaansen et al., 2009; Kuhbandner et al., 2010), joint actions (Godman, 2013; Pesquita et al., 2018), emotional disorders (Gjelsvik et al., 2018), and body psychotherapy (Röhricht et al., 2014).

Still, while in the relationship between two or more agents the involvement of the affective variable, even when uninvestigated, is intrinsically evoked (and extensively scrutinized by affective neuroscientists, Panksepp, 1998), in the case of the agent-object relationship the recognition of such engagement requires more specific care.

In laboratory-based studies, when dealing with an object and an observer, the practical opportunities that she is able to perceive and use (Gibson, 1979) have been mainly operationalized referring to visual manipulable properties of the object, as shape and orientation, associated with its canonical use (Tucker and Ellis, 1998). Progressively empirical research introduced, and manipulated, also the physical context, and the required responses, distinguishing between functional and volumetric gestures (Bub et al., 2008). Are these “affordances”? Strictly speaking no, as these accounts clash with direct perception, but they are undoubtedly elegant approaches suitable for outlining answers (also) to most questions of ecological psychology (for a masterly, unbiased, review, Chong and Proctor, 2020).

In light of the heated debate on affordances between philosophers and cognitive scientists, we propose to draw upon literature in both fields as our aim is twofold. (1) Exploring *the great absentee* of empirical investigations conducted so far: the affective dimension of perception-action coupling of our relationship with the physical context. To this end a clarification of the philosophical concept of “affective affordance” (henceforth AA: Griffiths and Scarantino, 2009; Hufendiek, 2016; Fuchs, 2017; Krueger and Colombetti, 2018) would be essential. (2) Specifying some criteria of definition for this construct and suggesting an analysis of AAs in its application to the individual human agent's practice—for our proposal to be not only theoretical, but suitable for experimental investigation, promoting a constructive dialogue between philosophy and empirical psychology.

The focus on the individual level is in no way intended to overshadow the need to examine AAs in relation to a larger-scale dimension of human experience (i.e., the distribution and historical accumulation of affective meanings in different communities, Goodwin, 2013). However, the latter is necessarily a subsequent level of analysis, since empirical investigation typically requires an incremental approach, even if the variables involved in a complex phenomenon interact non-linearly.

## The Subjective Dimension of Affordances

Affordances are perceived opportunities for action that arise out of the interaction between an embodied organism and its environment. These opportunities can be “either for good or ill” (Gibson, 1966, p. 285), meaning that when the agent perceives possibilities for action, she would directly perceive their good-ness or bad-ness in relation to her needs, motives, interests, and goals.

Although a valence-based approach could be consistent with the original theory of affordances (Gibson, 1966, 1979), a closer reading of Gibson's work might cast some doubt on this interpretation. Indeed, Gibson distinguishes the concept of affordance from valence-based constructs, such as “invitation character” and “demand character” (Reed and Jones, 1982; Kiverstein et al., 2019). Thus, whereas valence-based constructs serve to account for the subjective underpinnings of perceptual experience (e.g., affective states), *affordance* only refers to an invariant combination of factors that allows the agent to manipulate her environment despite the variability of the flux of perceptual stimuli (Gibson, 1979). This focus on invariants might be one of the reasons why philosophical research started to study the subjective (e.g., affective) dimension of affordances only in recent years (Rietveld, 2008; Gallagher, 2017; Dings, 2018; Krueger and Colombetti, 2018).

Yet, there is a body of experimental literature encouraging an inquiry into the subjective contextual features of *motor* affordances. By addressing evidence that shows how objects can elicit multiple affordances depending on the context and the task, Borghi and Riggio (2015) have proposed to distinguish between *stable* and *variable* affordances, deriving, respectively, from invariant objects properties and from more temporary objects characteristics. Shifting the focus to the agent-object *spatial* relation, Costantini et al. (2011) found that the emergence of affordance is modulated also by object distance, exactly by the *actual* object reachability, constrained by the *actual* functional capabilities of one's body (Ambrosini et al., 2012). Even language plays a role: for instance, action and observation verbs differently affect object affordance, in keeping with the proposal that language acts as a sort of filter (Borghi, 2012). Recent work has focused specifically on subjective valence: using the approach-avoidance paradigm, the stimulus-evaluation, in conjunction with the reference-frame (self/object), was shown to be critical in guiding behavior (Saraiva et al., 2013). Consistently, we believe that a systematic investigation on the role of subjective-affective components for the emergence of affordances is badly needed.

## The Likelihood of Affective Activation

The concept of AA was elaborated to accommodate the fact that “we perceive […] things as affording regulative opportunities to amplify, suppress, extend, enrich, and explore […] our affective experiences” (Krueger and Colombetti, 2018: 214). Meaning that environmental items—such as tools (e.g., musical instruments: Colombetti and Krueger, 2015), material objects (e.g., colored clothes: Colombetti and Krueger, 2015), and cultural artifacts (e.g., a rosary: Colombetti and Roberts, 2015)^1^—not only afford cognitive, motor, and functional actions but also shape affective components (e.g., bodily expressions and action tendencies) and processes (e.g., emotion regulation and enkinesthesia: Stuart, 2010, 2012, 2016).

These items of the environment may afford emotions due to the relation between the items' properties (e.g., material properties, associated cultural and social meanings: Bar and Neta, 2006; Malafouris, 2013) and the human agent's sensorimotor skills (Chemero, 2003, 2009), her mastery of social-cultural norms (Ramstead et al., 2016; Roche and Chainay, 2017; Veissière et al., 2019), as well as her affective abilities and states. In addition, in line with Gibson' concept of “nest of affordances” (Gibson, 1979) and with current enactive-ecological approaches (Rietveld and Kiverstein, 2014; Rietveld et al., 2018), AAs are components of complex niches of possibilities for action, which are more or less relevant in the agent's everyday experience depending on different factors (e.g., reliability and trustworthiness; Krueger and Colombetti, 2018).

Here we investigate these factors to better understand how some items of the environment become part of an AA relation and to lay the bases for future research. Considering the “nest-like” features of affordances and the pervasive influence of the agent's affective skills and states on perception (Barrett and Kensinger, 2010; Zadra and Clore, 2011; Pourtois et al., 2013; Niedenthal and Wood, 2019), one may indeed claim that *any* affordance relation instantiates some kind of affective action or reaction, therefore it should be considered as a full-fledged AA. To avoid a potential overextension of the construct we propose to integrate it with the notion of *likelihood* of affective activation, suggesting that it correlates with the details of object integration in the agent's practice.

Building on Schutte et al.' concept of emotional affordance as the likelihood of a situation eliciting emotional states and behaviors (Schutte et al., 2008), we suggest using AA to refer to relations with objects that are able to consistently solicit an emotional behavior over time, interpreting *integration* (Menary, 2009; Kirchhoff, 2014; Heersmink, 2015) as a means to predict whether an affordance-relation of the agent's practice is able to solicit an emotional behavior in a consistent and reliable manner. We use *integration* as a specification of the enactive concept of diachronic coupling, with the aim of identifying two intertwined dimensions that might influence the likelihood of affective activation: (i) the *level* of integration of an object in the agent's practice, and (ii) its *modality* of integration.

## Level and Modality of Integration

The *level* of integration expresses the quantitative aspect of AAs regarding the temporal dimension of the agent's practice: the more an agent interacts with that object, the higher its level of integration would be. This description of dimension (i) may be consistent with the conditions of agent-environment coupling elaborated in the literature on “extended” affectivity (Colombetti and Roberts, 2015) and it is useful to emphasize the importance of a quantifiable variable of integration: *frequency of exposure*^2^. The agent's exposure to an object has indeed been shown to positively correlate with the agent's trust in that object (Komiak and Benbasat, 2006), suggesting the introduction and manipulation of the variable “trust,” endorsed also by “extended” approaches to emotions. Support to this proposal comes (indirectly) from a study by Constable et al. (2011), who found that the automatic potentiation of action toward a graspable object is relatively strong for a self-decorated mug, used daily for 12–16 days, while it is abolished for an unfamiliar mug. This seems to point out that the action system is less sensitive to the potential for action toward objects that cannot be integrated in the agent's habitual affective practices. Hence, provided that the increased frequency of exposure might influence the agent's perception of the affective values of objects (Zajonc, 1968; Bornstein, 1989; Garcia-Marquez et al., 2016) and her expectations on their affective regulative effects, we suggest that proper AAs are instantiated by objects that have a significant level of integration in the agent's subjective practice.

The *modality* of integration expresses the qualitative aspect of AAs, and it can be used to specify the details of the agent's affective coupling with some objects, thus strengthening the theoretical connection between ecological approaches and the enactive conception of “extended” affective systems. Our suggestion is that for an object to be part of an “extended” affective system, the agent should have integrated it in her practices at some point in time according to an affective modality. This condition does not rule out the fact that an object may instantiate an AA also because of its functional properties. Still, it serves to distinguish two cases. First, the case in which the human agent interacts with an object in a mere functional way (e.g., Borghi et al., 2012) and still the object exerts an influence on the agent's affective states and behaviors, as a mug that the agent usually uses for drinking coffee. Second, the case in which an object solicits emotional states and behaviors because it is constitutive of a practice that is properly affective, as an old mug to which the agent is emotionally attached because it reminds her of her childhood. In our view, the latter case exemplifies the concept of AA in extended-enactive systems better than in the former. Indeed, in the former, the affective influence of the object on the agent seems to be causal: the coffee contained in the mug constrains the agent's affective states because of its bio-chemical properties. In the latter, this influence seems to be due to a constitutive affective relation built over time not only on the basis of the agent's recognition of the embodied regulatory effects of the object, but also on the basis of a more complex history of affective relations with it. Like in the former case, this affective relation involves physiological reactions due the agent's perceptual engagement with the object, but also a broader affective incorporation that pertains to the agent's self-narrative. This affective integration is indeed enabled by the agent's affective episodic and autobiographical memories that might be thought to be incorporated into an “extended” narrative self (Heersmink, 2017), which is not only diachronically shaped by the agent's habitual practices, but also by the relation that the objects manipulated in these practices entertain with the individual agent's affective history.

## Are Affective Affordances Entitled to Join Empirical Research? Let's Talk About It!

Considering these two dimensions of integration, we therefore suggest using AAs to refer to affordance-relations characterized by a high level of integration and by the modality of affective integration. This characterization of AAs emphasizes their context-sensitiveness and subjective dimension at the diachronic level.

The empirical analysis of the construct of AA certainly benefits from the progress achieved in the investigation of motor affordance and intersubjectivity, emphasizing its context-sensitiveness at the synchronic level. Laboratory research has investigated affordances using 2D, then 3D, images of objects, gradually introducing the variable *context* (Chong and Proctor, 2020). The kind of context scrutinized is not only physical-spatial, but also social and linguistic (Gianelli et al., 2013): (stable) affordances are in fact codified in language (Borghi, 2012; Borghi et al., 2013). A thorough understanding is also derived from the manipulation of the type of task (Scorolli and Borghi, 2015) as well as of the intention of the agent (Bub and Masson, 2010). These progressive improvements go in the direction of a more ecological setting. Yet, in the study of AAs it will be even more important to take into account the required (motor) response: discrete-binary responses (i.e., key presses) would not allow an accurate investigation of AAs and, more seriously, would not enable the planning phase of the movement to be analyzed separately from the on-line control phase, since the influence of each falls as the movement unfolds (Glover, 2004).

AAs are not properly visual properties (unlike those typically investigated across empirical literature on affordances), however they are conveyed (also) by vision: the re-adaptation of existing paradigms can therefore come to our aid, in particular the kinematic analysis of the temporal course of hand movement (Scorolli et al., 2015) toward known objects arranged in an everyday-like environment. From the testing of the temporal dynamics we expect to detect an effect of the AA specifically in the early kinematic events (roughly 35% of movement duration), since they reflect planning more than on-line control, and planning is a relatively slow process sensitive to semantic contents (Glover, 2004).

Restricting our exploration to an “isolated” object has been functional to highlight the novelty and the promising contribution of the construct. Future exploration will have to include multiple, also “social,” objects. Indeed, in everyday practice the object is encountered or even used with other objects. In case of functional-individual relation (e.g., a mug and a teabag), the object overbearingly asks for the complementary one; interestingly this request is affected by the Other's eye-gaze (both effects found in the grasping action component: Scorolli et al., 2014).

When addressing the different sources impacting the object's “affective load,” the overall model cannot finally overlook the weight of societal norms and roles (e.g., object ownership, Scorolli et al., 2018), and most importantly of the linguistic dimension. Language incorporates certain kinds of affordances (privileging function over manipulation: Masson et al., 2008), but it also constrains and is constrained by object affordances. With reference to existing kinematics paradigms, we would expect that AAs modulate, for instance, the weight of language in affecting visuo-motor transformations when reaching and grasping an object. In the case of linguistic labels conveying information on object intrinsic properties (e.g., size, Gentilucci et al., 2000), we would predict that their modulation of the motor response (in particular the grasping component) is weaker in the case of affectively charged objects.

## Author Contributions

MC and CS have made a substantial, direct and intellectual contribution to the work, and approved it for publication. All authors contributed to the article and approved the submitted version.

## Conflict of Interest

The authors declare that the research was conducted in the absence of any commercial or financial relationships that could be construed as a potential conflict of interest.

## Footnotes

^1^Objects, tools, and artifacts may instantiate affective processes on the basis of a different organization of “shaping factors.” For instance, with regard to cultural artifacts, the agent's mastery of cultural information may have a heavier weight on the affective relation with the item in comparison to what may happen in other cases, such as those involving objects that function as personalized affective mementos (Caravà, 2020). Here, for brevity, we will use “object” as an inclusive term to refer to different AA relations.

^2^In literature, *frequency of exposure* (implying interaction as well) and *familiarity* are used interchangeably. Although these two variables reasonably correlate, we hold it critical to distinguish them: familiarity is properly defined by qualitative aspects [see dimension (ii)]. The pencil I use 10 times a day to write the shopping list does have the same frequency of use as the pencil I use 10 times a day to write my diary, but their degree of familiarity strongly diverges. To mere experiences of exposure, familiarity adds the emotional dimension, characterized by a specific intensity and valence.
